# Associations of Whole Blood n-3 and n-6 Polyunsaturated Fatty Acids with Blood Pressure in Children and Adolescents – Results from the IDEFICS/I.Family Cohort

**DOI:** 10.1371/journal.pone.0165981

**Published:** 2016-11-02

**Authors:** Maike Wolters, Valeria Pala, Paola Russo, Patrizia Risé, Luis A. Moreno, Stefaan De Henauw, Kirsten Mehlig, Toomas Veidebaum, Denés Molnár, Michael Tornaritis, Claudio Galli, Wolfgang Ahrens, Claudia Börnhorst, on behalf of the IDEFICS and I.Family consortia

**Affiliations:** 1 Leibniz Institute for Prevention Research and Epidemiology - BIPS, Bremen, Germany; 2 Epidemiology and Prevention Unit, Department of Preventive and Predictive Medicine, Fondazione IRCCS Istituto Nazionale Dei Tumori, Milan, Italy; 3 Epidemiology and Population Genetics, Institute of Food Sciences, National Research Council, Avellino, Italy; 4 DiSFeB, Department of Pharmacological and Biomolecular Sciences, University of Milan, Milan, Italy; 5 GENUD (Growth, Exercise, Nutrition and Development) Research Group, Instituto Agroalimentario de Aragón (IA2), Instituto de Investigación Sanitaria Aragón (IIS Aragón), Centro de Investigación Biomédica en Red Fisiopatología de la Obesidad y Nutrición (CIBERObn), University of Zaragoza, Zaragoza, Spain; 6 Department of Public Health, Faculty of Medicine and Health Sciences, Ghent University, Ghent, Belgium; 7 Department of Public Health and Community Medicine, Institute of Medicine, Sahlgrenska Academy, University of Gothenburg, Gothenburg, Sweden; 8 National Institute for Health Development, Tallinn, Estonia; 9 National Institute of Health Promotion, University of Pécs, Pécs, Hungary; 10 Research and Education Institute of Child Health, Nicosia, Cyprus; 11 Institute of Statistics, Faculty of Mathematics and Computer Science, University of Bremen, Bremen, Germany; Inserm; Univ. Lille; CHU Lille, FRANCE

## Abstract

**Background:**

Polyunsaturated n-3 and n-6 polyunsaturated fatty acids (PUFA) are precursors of biologically active metabolites that affect blood pressure (BP) regulation. This study investigated the association of n-3 and n-6 PUFA and BP in children and adolescents.

**Methods:**

In a subsample of 1267 children aged 2–9 years at baseline of the European IDEFICS (Identification and prevention of dietary- and lifestyle-induced health effects in children and infants) cohort whole blood fatty acids were measured by a validated gas chromatographic method. Systolic and diastolic BP was measured at baseline and after two and six years. Mixed-effects models were used to assess the associations between fatty acids at baseline and BP z-scores over time adjusting for relevant covariables. Models were further estimated stratified by sex and weight status.

**Results:**

The baseline level of arachidonic acid was positively associated with subsequent systolic BP (β = 0.08, P = 0.002) and diastolic BP (β = 0.07, P<0.001). In thin/normal weight children, baseline alpha-linolenic (β = -1.13, P = 0.003) and eicosapentaenoic acid (β = -0.85, P = 0.003) levels were inversely related to baseline and also to subsequent systolic BP and alpha-linolenic acid to subsequent diastolic BP. In overweight/obese children, baseline eicosapentaenoic acid level was positively associated with baseline diastolic BP (β = 0.54, P = 0.005).

**Conclusions:**

Low blood arachidonic acid levels in the whole sample and high n-3 PUFA levels in thin/normal weight children are associated with lower and therefore healthier BP. The beneficial effects of high n-3 PUFA on BP were not observed in overweight/obese children, suggesting that they may have been overlaid by the unfavorable effects of excess weight.

## Introduction

Hypertension is a major public health issue in industrialized countries. Given the high prevalence of overweight and obesity in all age groups, already children are affected by elevated blood pressure (BP) [1–4]. Further, elevated childhood BP has been shown to predict elevated BP in adolescence and adulthood [5–7] and other adulthood cardiovascular diseases [8–10]. Serum and erythrocyte fatty acids (FA) have been suggested to be associated with BP in adults [11–13] although not all studies confirmed this association [14].

In particular, polyunsaturated fatty acids (PUFA) and their metabolites, the long-chain PUFA (LC PUFA) such as eicosapentaenoic acid (EPA, 20:5n-3) and docosahexaenoic acid (DHA, 22:6n-3) of the n-3 series and arachidonic acid (ARA, 20:4n-6) of the n-6 series have been linked to BP [11, 12, 14]. As precursors for the production of prostaglandins and thromboxanes with effects on vasodilation and platelet aggregation LC PUFA can affect BP [15]. Additionally, ARA and EPA are substrates for the cytochrome P450 (CYP450) catalyzed biosynthesis of metabolites of which the ARA metabolite 20-hydroxyeicosatetraenoic acid (20-HETE) has been shown to act as a vasoconstrictor. In contrast, epoxides of ARA like epoxyeicosatrienoic acids (EETs) and of n-3 LC PUFA like epoxyeicosatetraenoic acids seem to exert BP lowering effects, e.g. by increasing nitric oxide production and vasodilation [16, 17]. Beneficial effects of n-3 LC PUFA on BP have also been attributed to their role as competitors of n-6 LC PUFA in the biosynthesis of eicosanoids and lipid mediators including those catalyzed by CYP450 [18]. Additionally, 20-HETE and EETs are involved in renal tubular and vascular function that may affect BP [19–21].

Intervention studies with fish oil supplements or n-3 rich fatty fish showed a small blood pressure lowering effect [22]. Also a meta-analysis of randomized controlled trials with EPA and DHA supplementation concluded that n-3 LC PUFA lowers systolic BP (SBP) and in high doses also diastolic BP (DBP) [23]. However, data from observational studies and from the limited number of intervention studies in children are less consistent [18]. Three studies reported positive associations between childhood n-3 LC PUFA and BP. In Danish children, cross-sectional data indicated whole blood EPA to be positively associated with DBP in boys but not in girls [24] while in Danish adolescents DHA was positively associated with SBP [25]. In a Finnish cohort, the sum of serum cholesterylester n-3 PUFA in childhood was positively associated with BP in adulthood after 27 years in males but not in females [26]. Against the background of the presumed biological mechanisms and results of intervention studies in adults, the reported positive associations of n-3 PUFA and BP were unexpected. Therefore, this study investigates the cross-sectional and prospective associations between whole blood n-3 and n-6 PUFA and BP in a large cohort of European children.

## Methods

### Study group

In the IDEFICS (Identification and prevention of dietary- and lifestyle-induced health effects in children and infants) baseline survey (T0) in 2007/2008, a population-based sample of 16 228 children aged 2 to 9.9 years from eight European countries (Belgium, Cyprus, Estonia, Germany, Hungary, Italy, Spain, Sweden) was examined. Follow-up examinations were conducted two (T1) and six (T3, I.Family study) years later; the study design has been described in detail elsewhere [27, 28]. In brief, the IDEFICS/I.Family study comprises one of the largest prospective European child cohorts. The main focus of the IDEFICS study was to investigate diet- and lifestyle related disorders in young children with a focus on overweight and obesity. A further follow-up of the health status including 7105 children of the original IDEFICS cohort was performed in the I.Family study to explore the determinants of dietary and other lifestyle behaviors in these children and adolescents as they have grown older. A large set of anthropometric and clinical examinations was conducted and biosamples were taken as well as several questionnaires and interviews were collected. Lifestyle behaviors like dietary intake, physical activity and screen time of young children up to the age of 11 years were reported by parents whereas this information was self-reported by adolescents. Parents reported their own educational level and lifestyle behaviors like smoking in self-completion questionnaires. In a face-to-face interview the health conditions of the participating child and the family history of diseases were collected by trained interviewers.

Since in IDEFICS community- and setting-oriented intervention programs were implemented, in each country two mutually comparable areas have been allocated to either serve as intervention or control region [29].

Parents gave written informed consent for themselves and for their participating children. Children aged 12 years and over also gave written informed consent while small children gave oral consent prior to the start of the examinations. The participating centers in each country obtained ethical approval by the competent Institutional Review Boards prior to starting the study. These were the following ethics committees: Ethics Committee, University Hospital, Gent, Belgium; Cyprus National Bioethics Committee, Nicosia, Cyprus; Tallinn Medical Research Ethics Committee, Tallinn, Estonia; Ethics Committee of the University of Bremen, Bremen, Germany; Egészségügyi Tudományos Tanács, Pécs, Hungary; Azienda Sanitaria Locale Avellino Comitato Etico, Avellino, Italy; Regionala Etikprövningsnämnden i Göteborg, Gothenburg, Sweden; Comité Ético de Investigación Clínica de Aragón, Zaragoza, Spain. The examinations were conducted by trained field work teams. The authors of this manuscript had no contact to the participants.

**Fig 1:**
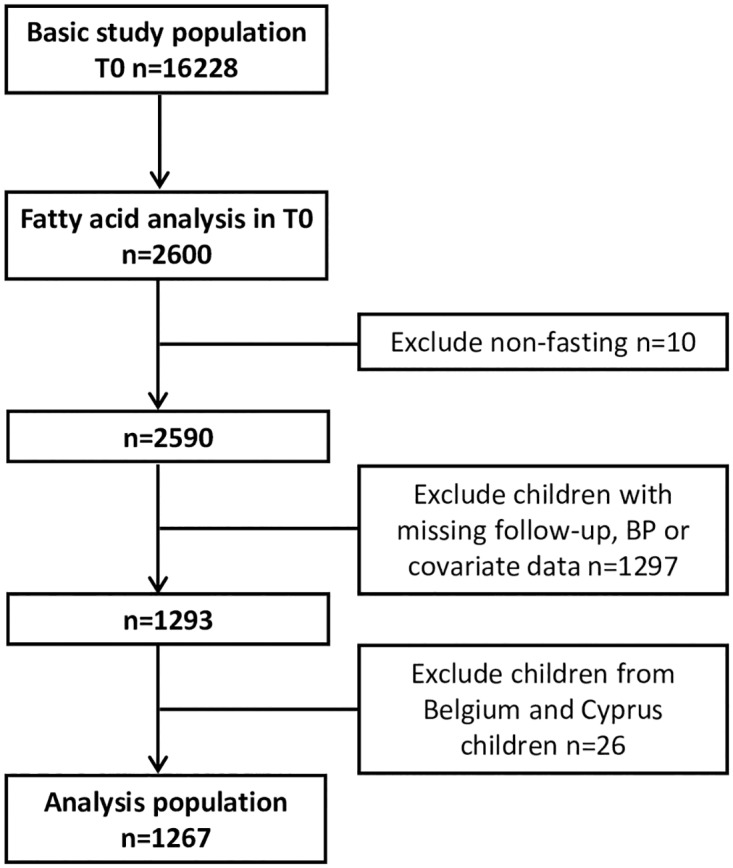
Flow chart of the inclusion and exclusion of IDEFICS/I.Family participants.

This study is based on a subsample of IDEFICS T0 participants with an oversampling of overweight and obese children [27]. FA profiles were analyzed in 2600 children of which 10 children were excluded because of blood drawing in a non-fasting state. Furthermore, only children with at least one follow-up measurement were included such that additional 1297 children had to be excluded due to missing BP, missing follow-up or missing covariate data. Because the remaining sample of children from Belgium and Cyprus consisted of only 15 and 11 children, respectively, these were not considered in the final analysis resulting in an analysis sample of 1267 children (Fig 1). The age range of the children was 2–9 years at T0, 4–11 years at T1 and 9–16 years at T3, respectively.

### Exposures

Blood was obtained by collecting a drop of blood from a fingertip or by venipuncture which was applied directly to a test strip prepared with butylated hydroxytoluene (BHT). FA in whole blood were separated and determined by gas-liquid chromatography after direct derivatisation to their methyl esters without prior extraction of total lipids from the samples in a central laboratory as previously described [30, 31]. This simple and rapid analysis method was validated by several laboratories [31–33]. Whole blood includes FA from all lipid classes and is representative for the total FA pool [32]. In the present analysis we used FA values of the n-3 PUFA alpha-linolenic acid (ALA, 18:3n-3), eicosapentaenoic acid and docosahexaenoic acid and the n-6 PUFA linoleic acid (LA, 18:2n-6), gamma-linolenic acid (GLA, 18:3n-6), dihomo-gamma-linolenic acid (DGLA, 20:3n-6) and arachidonic acid. FA are expressed as weight percentage of all FA detected (% wt/wt).

### Outcomes

SBP and DBP was measured at the right arm with a cuff of appropriate size for arm circumference after at least 5 minutes of rest in seated position according to a standardized protocol with an automated oscillometric device (Welch Allyn, Inc., 4200B-E2, Skaneateles Falls, NY, USA) [34]. Two recordings were taken, with a 2-min interval between each, plus a third measurement in case of a >5% difference between the first two readings. The mean value of the two measurements with the smallest difference was used as outcome measure. If the first and second measurement deviated by >5% and no third measurement value was obtained BP was set to missing. For SBP, DBP and BP classification to normal BP, pre-hypertension and hypertension, age, sex and height specific reference values from the US Department of Health and Human Services, National Institutes of Health (NIH, 2005) [35] were applied. SBP and DBP were further transformed to age-, sex- and height-specific z-scores according to the NIH references and were used as outcome variables in the subsequent analysis.

### Anthropometric data

Height of the children was measured to the nearest 0.1 cm with a calibrated stadiometer (Seca 225/213 stadiometer, Birmingham, UK). Body weight was assessed in fasting state in light underwear on a calibrated scale accurate to 0.1 kg (adapted Tanita BC 420 MA for children ≤6 years, BC 418 MA for children >6 years, Tanita Europe GmbH, Sindelfingen, Germany). BMI was calculated as weight [kg] divided by height [m] squared.

### Confounders

Potential confounders such as age (continuous), sex, country (6 categories: Estonia, Germany, Hungary, Italy, Spain, Sweden), maximum ISCED (International Standard Classification of Education [36]) level of parents (three categories: low level (ISCED 0,1,2); medium (ISCED 3,4); high (ISCED 5,6)) [36], parental smoking (5 categories, only assessed at T3: current daily smoker; current occasional smoker; ex-smoker; ex-smoker with missing pack years; never smoker), family history of hypertension (yes vs. no; as reported for biological parents/siblings at any of the three time points), BMI z-scores calculated based on Cole & Lobstein (2012) [37], height, birth weight and pubertal stage (pubertal, pre-pubertal, no information available; defined based on voice change in boys and first menstrual period in girls) were considered. To account for individual variations in the whole blood FA content depending on the FA distribution in lipid classes of plasma, lipoproteins and red blood cells of whole blood [32], we included weight percentage of total saturated (SFA) and total monounsaturated FA (MUFA) of total FA as covariates. A binary variable indicating control versus intervention regions was used to adjust for potential differences resulting from the intervention [29]. As indicator of dietary salt intake we used the weekly frequency of processed food consumption which includes Hamburger, hot dog, kebab, wrap, falafel, salty snacks such as savory pastries and fritters as well as cold cuts and ready to cook meat products. Television and computer time calculated as average screen time per week was also considered as a potential confounder.

Physical activity (PA) was measured objectively in a subset of children using the uniaxial Actigraph accelerometer (Actigraph MTI, model GT1M, Manufacturing Technology Inc., Fort Walton Beach, FL, USA) and the ActiTrainer which was the same technology as the Actigraph. We used time spent in moderate-to-vigorous physical activity (MVPA) according to the cut-points of Evenson et al. (2008) [38] including children with at least 3 accelerometer measurement days and at least 360 minutes of valid time per day as indicator of PA in sensitivity analyses.

### Statistical analyses

Mixed-effects models were used to assess the associations between the different FA measured at baseline and BP z-scores over time. These models provide a flexible tool to model repeated measurements data as they do not require individuals to be measured at the same ages and as subjects with a varying number of repeated measurements can be considered under a missing at random assumption[39]. They further allow for correlations among repeated measurements taken from the same subject. In the present analysis, all children with a baseline and at least one follow-up BP measurement were included (N = 1267; 1267 baseline measurements; 1069 T1 measurements and 667 T3 measurements). A variable indicating time since baseline was constructed to assess how associations change over time (takes the values 0 for the baseline survey, approx. 2 years for the first follow-up and approx. 6 years for the second follow-up). The model was built as follows:

$$\begin{array}{l} BP_{i,j}=\;(\beta_{0}+u_{i,0})+(\beta_{1}+u_{i,1})\;time\_since\_T0_{j}+\beta_{2}T0\_exposure_{i}\\ \;+\;\beta_{3}T0\_exposure_{i}\times time\_since\_T0_{i,j}+\beta_{covars}T0\_covariates_{i}\\ \;+\;\varepsilon_{i,j} \end{array}$$

*BP*_*i*,*j*_ denotes the outcome value of individual *i* measured at time *j* (*i = 1*,*…N* where *N* denotes the number of study subjects; *j = 0*,*1*,*2* denotes the survey wave), *β*_0_ denotes the intercept, *u*_*i*,0_ a random subject-specific intercept, *β*_1_ indicates the yearly change in average BP levels since baseline, *u*_*i*,1_ is a random subject-specific slope allowing BP to change differently in individuals over time, *β*_2_ is the effect of the T0 exposure on the outcome at T0, *β*_3_ describes the change per year in the effect of the FA exposure at baseline on BP over time and finally *β*_*covars*_ was used to indicate all effect estimates of the covariates included in the different models (e.g. if age and sex were included as covariates, *β*_*covars*_ would indicate the two effect estimates *β*_*age*_ and *β*_*sex*_ for age and sex). *ε*_*i*,*j*_ is the error term for individual *i* at time *j*. An unstructured covariance matrix was selected for the random effects, i.e. each variance/covariance could take the value that the data demand.

For all exposures and the two outcomes (SBP z-score, DBP z-score), a basic model adjusting only for age (continuous), sex, height, country and intervention vs. control region (dummy variable) was estimated in a first step. In a second step, the following covariates were added to the basic model: maximum ISCED level of parents, parental smoking, family history of hypertension, BMI z-score, pubertal status, birth weight, weight percentage of the sum of SFA and of MUFA of total FA, weekly frequency of processed food consumption and weekly hours of screen time (fully adjusted model). Due to the significant interaction between EPA and weight status, models were further estimated stratified by sex and stratified by BMI category (thin/normal weight vs. overweight/obese; categorization according to Cole & Lobstein (2012) [37]).

In the subgroup with available accelerometer data (N = 1035), the fully adjusted model was additionally adjusted for minutes in MVPA.

All analyses were performed using SAS^®^ statistical software version 9.3 (SAS Institute, Inc., Cary, NC). All models were run using SAS Proc Mixed.

*P* values were not adjusted for multiple comparisons because of the explorative nature of this study. A *P* value of 0.01 was used as the level of statistical significance to account at least partially for multiple testing.

## Results

The baseline characteristics of the study population are presented in Table 1. Of the girls, 20.7% and of the boys 20.2% were classified as pre-hypertensive or hypertensive. BP z-scores were higher in girls than in boys. Weight percentages of PUFA were similar among BP categories. 42% of the children were overweight or obese with slightly more boys than girls being classified as obese. BMI z-scores were higher whereas birth weight was slightly lower in children with normal BP compared to hypertensive children. Among the 220 children with a family history of hypertension, 27.3% were pre-hypertensive or hypertensive while this was true for only 19.0% of the 1047 children without family history of hypertension. 21.5% of children whose parents were current smokers were pre-hypertensive or hypertensive whereas this applied to 18.0% of children with never or ex-smoker parents.

**Table 1:** Baseline characteristics of the study population by blood pressure category^1^ and sex.

		All	Normal BP	Pre-hypertension	Hypertension	Girls	Boys
Sex, n (%)	Girls	1267	494 (79.3)	70 (11.2)	59 (9.5)	623 (49.2)	644 (50.8)
	Boys		514 (79.8)	78 (12.1)	52 (8.1)		
Age, years	Mean (SD)	6.25 (1.7)	6.27 (1.7)	5.93 (1.9)	6.49 (2.0)	6.26 (1.7)	6.24 (1.8)
	2-<6 years, n (%)	509 (40.2)	406 (79.8)	66 (13.0)	37 (7.3)	253 (49.7)	256 (50.3)
	6-<10 years, n (%)	758 (59.8)	602 (79.4)	82 (10.8)	74 (9.8)	370 (48.8)	388 (51.2)
BP, Mean (SD)	SBP (mmHg)	101.4 (9.60)	98.8 (7.57)	107.4 (7.88)	117.2 (9.43)	101.0 (9.52)	101.8 (9.67)
	DBP (mmHg)	63.6 (6.52)	61.7 (5.21)	68.9 (4.44)	74.1 (5.58)	63.8 (6.46)	63.4 (6.58)
	SBP, z-score	0.40 (0.80)	0.15 (0.60)	1.03 (0.54)	1.87 (0.64)	0.46 (0.82)	0.36 (0.78)
	DBP, z-score	0.64 (0.59)	0.46 (0.45)	1.20 (0.38)	1.56 (0.57)	0.66 (0.59)	0.62 (0.59)
PUFA, Mean (SD), % wt/wt^2^	18:2n-6, LA	17.8 (1.96)	17.9 (1.94)	17.6 (1.94)	17.9 (2.17)	18.0 (1.99)	17.7 (1.93)
	18:3n-6, GLA	0.23 (0.09)	0.23 (0.09)	0.22 (0.08)	0.23 (0.09)	0.22 (0.09)	0.23 (0.09)
	20:3n-6, DGLA	1.23 (0.26)	1.23 (0.27)	1.20 (0.24)	1.22 (0.28)	1.20 (0.26)	1.25 (0.27)
	20:4n-6, ARA	7.47 (1.31)	7.46 (1.30)	7.57 (1.29)	7.40 (1.40)	7.38 (1.34)	7.55 (1.27)
	18:3n-3, ALA	0.20 (0.09)	0.20 (0.09)	0.19 (0.09)	0.21 (0.09)	0.20 (0.09)	0.20 (0.08)
	20:5n-3, EPA	0.28 (0.12)	0.27 (0.12)	0.27 (0.11)	0.30 (0.13)	0.28 (0.13)	0.27 (0.11)
	22:6n-3, DHA	1.23 (0.44)	1.23 (0.44)	1.23 (0.40)	1.24 (0.44)	1.21 (0.44)	1.26 (0.44)
SFA, Mean (SD), % wt/wt	sum	44.4 (1.89)	44.4 (1.92)	44.5 (1.77)	44.3 (1.87)	44.4 (1.96)	44.5 (1.83)
MUFA, Mean (SD), % wt/wt	sum	25.2 (2.35)	25.2 (2.32)	25.2 (2.49)	25.2 (2.48)	25.3 (2.42)	25.1 (2.29)
Family history of hyperten-sion, n (%)	no	1047 (82.6)	848 (81.0)	117 (11.2)	82 (7.8)	517 (49.4)	530 (50.6)
	yes	220 (17.4)	160 (72.7)	31 (14.1)	29 (13.2)	106 (48.2)	114 (51.8)
ISCED level, n (%)	Level 0, 1, 2	148 (11.7)	117 (79.1)	17 (11.5)	14 (9.5)	69 (46.6)	79 (53.4)
	Level 3, 4	664 (52.4)	541 (81.5)	70 (10.5)	53 (8.0)	320 (48.2)	344 (51.8)
	Level 5, 6	455 (35.9)	350 (76.9)	61 (13.4)	44 (9.7)	234 (51.4)	221 (48.6)
Height, Mean (SD)	cm	119.2 (12.5)	119.4 (11.8)	116.8 (13.8)	120.9 (15.5)	118.7 (12.1)	119.7 (12.7)
BMI, Mean (SD)	kg/m^2^	17.9 (3.25)	17.6 (3.08)	18.0 (3.29)	20.2 (3.74)	17.7 (3.08)	18.0 (3.40)
BMI, Mean (SD)^2^	z-score	0.98 (1.30)	0.90 (1.29)	0.97 (1.34)	1.75 (1.15)	0.96 (1.23)	1.00 (1.38)
BMI category, n (%)^3^	Thin	76 (6.0)	64 (84.2)	10 (13.2)	2 (2.6)	31 (40.8)	45 (59.2)
	Normal weight	659 (52.0)	551 (83.6)	73 (11.1)	35 (5.3)	330 (50.1)	329 (49.9)
	Overweight	281 (22.2)	215 (76.5)	36 (12.8)	30 (10.7)	142 (50.5)	139 (49.5)
	Obese	251 (19.8)	178 (70.9)	29 (11.6)	44 (17.5)	120 (47.8)	131 (52.2)
Birth weight, Mean (SD)	g	3373 (545)	3376 (548)	3367 (571)	3352 (471)	3303 (532)	3440 (548)
Parental smoking, n (%)^2^	Never smoker	336 (26.5)	272 (81.0)	39 (11.6)	25 (7.4)	174 (51.8)	162 (48.2)
	Current smoker	181 (14.3)	142 (78.5)	27 (14.9)	12 (6.6)	91 (50.3)	90 (49.7)
	Ex-smoker	142 (11.2)	120 (84.5)	14 (9.9)	8 (5.6)	60 (42.3)	82 (57.7)
	Missing information	608 (48.0)	474 (78)	68 (11.2)	66 (10.9)	298 (49.0)	310 (51.0)
Consumption frequency of processed food, Mean (SD)	times per week	5.4 (4.9)	5.3 (4.9)	5.9 (5.5)	5.8 (4.4)	5.2 (4.6)	5.6 (5.3)
Time spent with audiovisual media, Mean (SD)	hours per week	12.1 (7.9)	12.1 (8.0)	12.2 (7.5)	12.4 (6.8)	10.8 (6.5)	13.4 (8.9)

^1^National Institutes of Health 2005

^2^As only selected PUFA are shown, the sum of SFA, MUFA and PUFA does not sum up to 100%

^3^Cole & Lobstein 2012

ALA, alpha-linolenic acid; ARA, arachidonic acid; BMI, body mass index; BP, blood pressure; DBP, diastolic blood pressure; DHA, docosahexaenoic acid; DGLA, dihomo-gamma-linolenic acid; EPA, eicosapentaenoic acid; GLA, gamma-linolenic acid; ISCED, International Standard Classification of Education; LA, linoleic acid; MUFA, monounsaturated fatty acids; PUFA, polyunsaturated fatty acids; SBP, systolic blood pressure; SD, standard deviation; SFA, saturated fatty acids

Table 2 shows the associations of the different PUFA with SBP and DBP z-scores at baseline, after two years and after six years of follow-up. In the fully adjusted model, baseline ARA was positively associated with subsequent SBP and DBP indicating that 1 unit increase of ARA was associated with a 0.08 units higher SBP and a 0.07 units higher DBP z-score after 6 years.

**Table 2:** Associations of polyunsaturated fatty acids with systolic and diastolic blood pressure z-scores at baseline, after 2 years and after 6 years of follow-up estimated based on fully adjusted mixed-effects models^1^.

Fatty acid	Time since baseline years	SBP		DBP	
		β	*P* value	β	*P* value
18:2n-6, LA	0	-0.02	0.212	-0.02	0.024
	2	-0.02	0.198	-0.02	0.086
	6	-0.02	0.372	0.00	0.839
18:3n-6, GLA	0	-0.17	0.453	-0.16	0.324
	2	-0.09	0.655	0.04	0.739
	6	0.07	0.820	0.45	0.046
20:3n-6, DGLA	0	0.02	0.819	-0.06	0.346
	2	0.04	0.638	0.01	0.875
	6	0.08	0.521	0.15	0.071
20:4n-6, ARA	0	0.04	0.100	0.01	0.682
	2	0.05	0.012	0.03	0.050
	6	0.08*	0.002	0.07**	0.000
18:3n-3, ALA	0	-0.25	0.362	-0.01	0.971
	2	-0.36	0.163	-0.18	0.338
	6	-0.59	0.136	-0.53	0.059
20:5n-3, EPA	0	-0.26	0.158	0.11	0.382
	2	-0.31	0.073	-0.07	0.568
	6	-0.41	0.122	-0.42	0.019
22:6n-3, DHA	0	-0.11	0.048	-0.01	0.869
	2	-0.10	0.064	-0.02	0.641
	6	-0.06	0.385	-0.04	0.466

^1^Model, adjusted for age, sex, height, country and intervention vs. control region, maximum ISCED level of parents, parental smoking, family history of hypertension, BMI z-score, pubertal status, birth weight, weight percentage of the sum of SFA and of MUFA of total FA, weekly frequency of processed food consumption and weekly hours of screen time.

*P<0.01;

**P<0.001

ALA, alpha-linolenic acid; ARA, arachidonic acid; BMI, body mass index; DBP, diastolic blood pressure; DHA, docosahexaenoic acid; DGLA, dihomo-gamma-linolenic acid; EPA, eicosapentaenoic acid; GLA, gamma-linolenic acid; ISCED, International Standard Classification of Education; LA, linoleic acid; MUFA, monounsaturated fatty acids; SBP, systolic blood pressure; SFA, saturated fatty acids

Results stratified by BMI category revealed that associations differ by baseline BMI (Table 3). The observed positive association between ARA and BP pointed to the same direction in all subgroups. In thin/normal weight children, inverse associations of baseline ALA and EPA with SBP z-score at baseline and after 2 years of follow up and of baseline ALA with subsequent DBP z-score were observed. Children classified as overweight/obese showed a positive association of baseline EPA with DBP z-score at baseline. For instance, one unit increase of baseline EPA was associated with 0.85 units lower SBP z-score at baseline in thin/normal weight children but with 0.54 higher DBP z-score at baseline in overweight/obese children.

**Table 3:** Associations of fatty acids with systolic and diastolic blood pressure z-scores at baseline, after two years and after six years of follow-up stratified by BMI category^1^.

Fatty acid	Time since baseline years	SBP, thin / normal weight n = 735		SBP, overweight / obese n = 532		DBP, thin / normal weight n = 735		DBP, overweight / obese n = 532	
		β	*P* value	β	*P* value	β	*P* value	β	*P* value
18:2n-6, LA	0	0.01	0.565	-0.04	0.082	0.00	0.921	-0.04	0.012
	2	0.00	0.900	-0.03	0.166	0.01	0.671	-0.03	0.035
	6	-0.01	0.537	-0.01	0.820	0.01	0.425	-0.01	0.521
18:3n-6, GLA	0	-0.70	0.018	0.31	0.392	-0.40	0.067	0.06	0.809
	2	-0.58	0.028	0.36	0.241	-0.21	0.254	0.19	0.346
	6	-0.36	0.413	0.48	0.338	0.17	0.589	0.44	0.182
20:3n-6, DGLA	0	-0.01	0.923	0.00	0.982	-0.15	0.080	0.00	0.973
	2	0.01	0.928	0.03	0.837	-0.11	0.140	0.09	0.312
	6	0.05	0.748	0.07	0.679	-0.04	0.742	0.26	0.036
20:4n-6, ARA	0	0.02	0.582	0.04	0.255	-0.01	0.788	0.01	0.673
	2	0.03	0.197	0.05	0.112	0.01	0.478	0.03	0.159
	6	0.07	0.043	0.08	0.079	0.05	0.045	0.08	0.015
18:3n-3, ALA	0	-1.13*	0.003	0.49	0.250	-0.68	0.018	0.48	0.118
	2	-1.03*	0.004	0.17	0.686	-0.75*	0.004	0.20	0.496
	6	-0.85	0.091	-0.48	0.442	-0.89	0.016	-0.38	0.374
20:5n-3, EPA	0	-0.85*	0.001	0.38	0.148	-0.29	0.089	0.54*	0.005
	2	-0.71*	0.007	0.09	0.723	-0.36	0.024	0.23	0.208
	6	-0.42	0.238	-0.50	0.198	-0.51	0.029	-0.40	0.179
22:6n-3, DHA	0	-0.19	0.011	-0.03	0.786	-0.04	0.392	0.04	0.525
	2	-0.14	0.039	-0.05	0.559	-0.06	0.201	0.03	0.596
	6	-0.05	0.580	-0.09	0.412	-0.09	0.166	0.01	0.889

^1^Model adjusted for age, sex, height, country, intervention vs. control region, maximum ISCED (International Standard Classification of Education) level of parents, parental smoking, family history of hypertension, BMI z-score, pubertal status, birth weight, weight percentage of the sum of SFA and of MUFA of total FA, weekly frequency of processed food consumption and weekly hours of screen time.

*P<0.01

ALA, alpha-linolenic acid; ARA, arachidonic acid; BMI, body mass index; BP, blood pressure; DBP, diastolic blood pressure; DHA, docosahexaenoic acid; DGLA, dihomo-gamma-linolenic acid; EPA, eicosapentaenoic acid; GLA, gamma-linolenic acid; ISCED, International Standard Classification of Education; LA, linoleic acid; MUFA, monounsaturated fatty acids; PUFA, polyunsaturated fatty acids; SBP, systolic blood pressure; SFA, saturated fatty acids

Table 4 shows the associations stratified by sex which confirms the positive association of baseline ARA with subsequent DBP in boys though not reaching significance in girls (p = 0.010). In boys, baseline GLA was positively associated with subsequent DBP indicating that 1 unit increase in GLA was associated with a 0.78 unit higher DBP z-score.

**Table 4:** Associations of fatty acids with systolic and diastolic blood pressure z-scores at baseline, after two years and after six years of follow-up stratified by sex^1^.

Fatty acid	Time since baseline years	SBP, girls n = 623		SBP, boys n = 644		DBP, girls n = 623		DBP, boys n = 644	
		β	*P* value	β	*P* value	β	*P* value	β	*P* value
18:2n-6, LA	0	-0.01	0.750	-0.03	0.131	-0.02	0.281	-0.03	0.055
	2	-0.01	0.516	-0.02	0.244	-0.02	0.212	-0.01	0.325
	6	-0.02	0.336	-0.01	0.791	-0.02	0.299	0.01	0.390
18:3n-6, GLA	0	-0.29	0.410	0.04	0.903	-0.44	0.078	0.05	0.818
	2	-0.28	0.373	0.19	0.472	-0.25	0.240	0.29	0.087
	6	-0.25	0.619	0.51	0.244	0.14	0.687	0.78*	0.009
20:3n-6, DGLA	0	0.03	0.841	0.00	0.968	-0.09	0.338	-0.06	0.512
	2	0.05	0.665	0.00	0.983	-0.01	0.911	0.01	0.912
	6	0.10	0.553	0.02	0.918	0.15	0.206	0.15	0.204
20:4n-6, ARA	0	0.01	0.677	0.06	0.041	0.01	0.601	0.00	0.987
	2	0.04	0.216	0.06	0.024	0.03	0.141	0.03	0.180
	6	0.09	0.024	0.07	0.071	0.07	0.010	0.08*	0.007
18:3n-3, ALA	0	-0.53	0.105	-0.19	0.655	-0.43	0.065	0.05	0.882
	2	-0.88	0.082	-0.21	0.733	-0.69	0.059	-0.40	0.336
	6	0.03	0.841	0.00	0.968	-0.09	0.338	-0.06	0.512
20:5n-3, EPA	0	-0.42	0.048	0.01	0.975	-0.14	0.342	0.50	0.044
	2	-0.36	0.066	-0.18	0.554	-0.29	0.054	0.29	0.180
	6	-0.25	0.429	-0.56	0.169	-0.59	0.019	-0.14	0.626
22:6n-3, DHA	0	-0.11	0.217	-0.11	0.178	0.01	0.884	-0.04	0.498
	2	-0.07	0.336	-0.10	0.142	-0.01	0.889	-0.04	0.402
	6	-0.01	0.942	-0.10	0.280	-0.04	0.603	-0.05	0.459

^1^Model adjusted for age, height, country, intervention vs. control region, maximum ISCED (International Standard Classification of Education) level of parents, parental smoking, family history of hypertension, BMI z-score, pubertal status, birth weight, weight percentage of the sum of SFA and of MUFA of total FA, weekly frequency of processed food consumption and weekly hours of screen time.

*P<0.01

ALA, alpha-linolenic acid; ARA, arachidonic acid; BMI, body mass index; BP, blood pressure; DBP, diastolic blood pressure; DHA, docosahexaenoic acid; DGLA, dihomo-gamma-linolenic acid; EPA, eicosapentaenoic acid; GLA, gamma-linolenic acid; ISCED, International Standard Classification of Education; LA, linoleic acid; MUFA, monounsaturated fatty acids; PUFA, polyunsaturated fatty acids; SBP, systolic blood pressure; SFA, saturated fatty acids

In the subgroup analyses with additional adjustment for MVPA the overall trends of associations remained unaltered (data not shown).

## Discussion

In our large sample of European children, we observed that high blood levels of ARA are positively associated with subsequent SBP and DBP z-scores indicating a small detrimental effect of high ARA on BP. ALA and EPA showed a beneficial effect on BP in the subgroup of thin/normal weight children in contrast to the results of previous observational studies investigating childhood blood n-3 PUFA (EPA and/or DHA) and BP [24–26, 40]. However, in overweight/obese children an unfavorable effect of EPA was observed. Thus, this study shows that already in children the weight status seems to modify the inverse association of n-3 FA with BP.

### n-6 PUFA

The adverse effect of high ARA on BP observed in our study confirms previous results in adults [41, 42] which to our knowledge have not been shown in children before. In contrast, in a Finnish cohort childhood serum cholesterylester levels of ARA were inversely associated with the BP after 27 years of follow-up but fat intake in Finland has strongly changed over time [26] and is probably different from our study population. Additionally, other factors may play a more important role after this long time period. ARA metabolites like prostaglandins and 20-HETE have vasoconstrictive, and thus blood pressure increasing, effects [15–17]. Additionally, ARA is a reliable indicator for animal-derived foods because the key desaturase steps in the conversion of LA to ARA, delta-6 desaturase (D6D) and delta-5 desaturase (D5D), are inhibited by high concentrations of the substrate [15]. Thus, high blood ARA reflects a higher consumption of animal-derived foods with high intake of animal fats. Such a dietary pattern has also been shown to be associated with elevated BP because of the corresponding high sodium intake [43]. We observed the association of baseline ARA with BP after 6 years of follow up. This may indicate that the adverse effects of the dietary pattern may become stronger with increasing age as dietary patterns have been shown to track from childhood to adolescence [44].

As observed in boys in our study, a positive association of GLA with BP was also found in Chinese and Western Alaskan Native adults [13, 45]. GLA is generated from LA by D6D and can—after being elongated to DGLA—further be desaturated by D5D to ARA. As D6D activity is higher and D5D activity is lower in male subjects [46, 47], the positive association in boys may be explained by sex differences in desaturase activity.

### n-3 PUFA

Our results indicate a protective role of ALA and EPA against hypertension in thin/normal weight children whereas in overweight/obese children high EPA seemed to affect DBP unfavourably. Beneficial effects of n-3 PUFA on BP have been shown in several intervention studies investigating the effect of supplemental FA intake [48–50] and in observational studies investigating dietary intake [8, 23, 51–53]. While observational studies investigating the association of n-3 PUFA blood levels and BP showed beneficial effects in adults [11, 42], results in children and adolescents were not consistent and indicated unfavorable effects [24, 25, 40]. In a Danish cross-sectional study including 8–11 year old children, whole-blood EPA was associated with a 2.7 mmHg higher DBP per weight% EPA in boys but not in girls [24]. Also in a large Finnish study an unexpected positive association of childhood cholesterylester EPA with BP after 27 years was observed indicating a stronger association in men than in women [26]. We did not observe sex-specific but a weight status-specific disparity in the n-3 PUFA-BP association in our study indicating a protective role of n-3 PUFA only in thin/normal weight children. Thus, the unfavorable effects of a high BMI and an altered n-3 PUFA status may have overlaid the protective effects of n-3 PUFA on BP. In line with our results, in Alaskan Native adults an inverse association of a marine food biomarker in blood and BP was observed in non-obese but not in obese subjects. The interaction of weight status and the biomarker was only significant for DBP but not for SBP [54]. In our analysis group, a high proportion of children (42%) were overweight or obese. Already in children, BMI has a strong independent blood pressure increasing effect [55, 56], and thus in our study sample obese children were more often hypertensive than thin or normal weight children. Additionally, overweight and obese children enter puberty usually earlier than thin or normal weight children [57, 58]. As BP increases with puberty [59, 60] and early puberty has shown to be a risk factor for later high BP [57], this may have also contributed to higher BP in overweight/obese children. Also, inflammatory cytokines and adipocytokines from adipose tissue in overweight and obese subjects were shown to increase blood pressure [61, 62] such that obesity-related effects on BP may have overlaid the BP lowering effects of n-3 PUFA observed in thin/normal weight children.

Data on the influence of BMI on blood n-3 LC PUFA concentrations are inconsistent [40, 63] whereas lower estimated D5D activity has consistently been observed in obese subjects compared to normal weight subjects indicating a less effective last desaturation step to ARA and EPA, respectively [63, 64]. Therefore, obese children may synthesize less EPA from its precursor. However, in our study, EPA percentages were slightly higher in overweight/obese children compared to thin/normal weight children (data not shown). This may have resulted from a higher total energy intake and a dietary pattern with high consumption of animal-derived foods including fish with high n-3 LC PUFA as also ARA percentages were higher in the upper BMI categories reflecting higher consumption of animal-derived fats as discussed before and as shown for overweight/obese subjects in previous studies [65–67].

Higher dietary EPA intake may be required to achieve BP lowering effects, particularly regarding DBP, as shown in a meta-analysis on the effects of fish oil or n-3 LC PUFA supplements on BP [23]. As children and adolescents generally do not meet the recommended dietary intake of n-3 LC PUFA [68], an increase of dietary EPA may beneficially influence BP.

According to a recent meta-analysis of prospective cohort studies in adult populations higher circulating DHA levels may prevent from elevated blood pressure [69]. In agreement with this, our data point to an inverse association of DHA with SBP in thin/normal weight children at baseline though the effect estimate did not reach statistical significance. In contrast, two smaller cross-sectional studies showed that whole-blood DHA was positively associated with DBP in 8–11 year old boys [40] and that erythrocyte DHA was positively related with SBP in 17 year old adolescents [25].

In our analysis, we used "time since baseline" (instead of age) as the main time axis in order to assess how associations between the FA exposures and BP change over time. However, when using age as the main time axis instead, we found that the association between ALA and SBP decreases with age whereas associations between ARA and DHA with BP increase with age.

### Limitations and strengths

A limitation of our study is that the repeated blood pressure measurements were taken only on a single occasion at each survey wave whereas confirmation of BP values on repeated visits or even ambulatory 24-hour monitoring is recommended for the diagnosis of hypertension. Therefore intra-individual variability which is well documented in children could not be considered and may have led to an overestimation of BP values [35, 70].

We adjusted only partially for multiple testing by choosing a significance level of 0.01 but no formal multiple testing correction was performed.

As detailed dietary intake data was only available in a smaller subgroup and would not have reliably reflected dietary salt intake, we used the intake frequency of processed foods as a proxy for sodium intake because processed foods have shown to be the main source of salt [71].

The large analysis group of >1200 children from 6 European countries as well as the longitudinal design and detailed phenotyping of the children are important strengths of our study. In all countries standardized protocols for data assessment, measurements and biosampling were applied at all survey waves and adherence to protocols was verified by a central quality control.

## Conclusions

Our results suggest that ARA adversely affects BP already in children and may contribute to the development of hypertension whereas n-3 PUFA exert BP lowering effects in thin and normal weight children. In overweight and obese children the detrimental effects of increased weight status may have overlaid the protective effects of n-3 fatty acids such that an unfavorable relation of EPA and DBP became apparent in this subgroup. Higher EPA, and especially higher DHA, combined with a healthier food pattern might be required to lower BP in both, thin/normal weight and overweight/obese children. Health interventions need to consider effect modification by weight status and should focus particularly on overweight and obese children who are more susceptible to elevated BP despite high n-3 PUFA levels.
